# Animal Welfare Certification Schemes in a Knowledge Society: A Fair Transition from Inputs to Outputs as a Driver of Animal Empowerment

**DOI:** 10.3390/ani15192854

**Published:** 2025-09-30

**Authors:** Antoni Dalmau

**Affiliations:** Animal Welfare Program, Institute of Agrifood Research and Technology (IRTA), Veinat de Sies S/N. Monells, 17121 Girona, Spain; antoni.dalmau@irta.cat

**Keywords:** animal welfare, animal-based indicators, assessment, consumer, emotions, Five Dominions, Five Freedoms, live worth living

## Abstract

**Simple Summary:**

Societal values and the concept of animal welfare are closely linked. Even when the first scientific definitions of animal welfare appeared in the United Kingdom, they did so in a society beginning to shift from the strict conservative values of the 1950s to the spirit of freedom embraced by the younger generations of the 1960s. These shifts ultimately created the conditions for the first definition of animal welfare, known as the Five Freedoms. In recent decades, European society has moved toward a greater connection between people and their own emotions, as well as the emotions of others, including those of animals. This has led to changes in the definition of animal welfare, with the introduction of new concepts such as a “Life Worth Living” or the Five Domains. However, most commercially available animal welfare certification systems are based on an outdated view of welfare, in which the animal is not assessed, but rather what the human provides or does for it is measured. This approach needs to be reconsidered to consider the interests and valence of animals.

**Abstract:**

Although concern for animal welfare may have been linked to humans since the domestication of livestock, the term itself first appeared in the United Kingdom in the 1960s. The emergence of the concept of animal welfare occurred in a society undergoing a clear transition from patriarchal to emancipatory values based on the concept of freedom. However, coinciding with the recognition of animals as sentient beings in the EU and the emergence of concepts such as a “Life Worth Living”, the Five Freedoms were complemented. In fact, the values of a knowledge society—through autonomy, justice, and equality—create the conditions for a society more connected to its emotions. This entire movement culminated in an updated and complementary definition called “the Five Domains,” in which the mental states of animals and their emotions are essential. However, in the meantime, the market is dominated by several animal welfare certification schemes that focus on inputs (what humans provide) rather than outcomes (animal-based indicators), reflecting an anthropocentric perspective that does not consider the actual experiences of animals from farm to farm. In a knowledge society, where emotions are so important, this approach will be considered unacceptable someday.

## 1. Introduction

Animal welfare became a topic of scientific interest in the 1960s, when the concept was first introduced to consider the conditions of farm animals. However, the emergence of the topic did not occur in isolation, disconnected from the society in which it arose. On the contrary, it appeared and grew, accompanied by two intrinsic forces. Firstly, scientific knowledge has advanced in recognizing animals’ capacity to feel emotions, among other abilities. Secondly, the society surrounding these scientists was undergoing a transition from a very rigid social hierarchy, with unquestionable respect for authority, to values of self-expression. This manuscript reviews how the evolution of human and social values has shaped the way farm animals are viewed and treated from their domestication to the present day. This has affected the definition of animal welfare by itself, from the Five Freedoms to the Five Domains, which can be better explained in relation to the transition from societies based on agricultural (historically patriarchal) values to knowledge societies, in which connection with one's own emotions and those of others is essential. However, as will be discussed in the Discussion Section, there is an asynchrony in this whole process, that is, in how animal welfare reaches the market and consumers through animal welfare certification schemes. Since, instead of empowering animals through the use of animal-based indicators (animal welfare outcomes), as is expected in a knowledge society, priority is given to production-system classification and input-based checklists that, in most cases, do not take into account the actual life experiences of animals.

## 2. From Domestication in the Mediterranean Region to the UK in the 1960s

Humans (*Homo sapiens*) have a history of 200,000 years [1]. For the first 190,000 years, we were nomadic hunter-gatherers, but after the last Ice Age (the Wurm Ice Age), most individuals of the species transitioned from nomadism to sedentarism, primarily because environmental conditions allowed for the domestication of plants and animals [2]. This occurred during the Neolithic period, between 12,000 and 2000 BC [3]. This revolution during the Neolithic period created the concept of possession and surpluses of products such as crops. This, in turn, led to trade to manage these surpluses. As a consequence, written language emerged as a way to record property and commercial exchanges. Property created the need to maintain a lineage to ensure its transfer between generations [4], and this was achieved through the recognition of paternity. Nature was privatized, and with it the means of production, such as land, animals, and human beings—that is, crops, livestock, slaves, women, and children [5]. The desire to preserve possessions (land and animals) from generation to generation led men to try to ensure that women would bear only their sons and that their offspring would inherit their property. This materialized not only in the central importance of paternity, which in earlier societies was not as important [6], but also in the emergence of something new, i.e., marriage. The anthropologist Claude Lévi-Strauss defined marriage at that time as a reciprocal alliance between men, where women were not part of the marriage, but rather its objects [7], as were animals and other possessions. The distribution of labor between the sexes that had characterized earlier hunter-gatherer societies changed to give way to a society in which men owned both the house (husband) and the herds (husbandry). Since these herds were the main food source, a patrilocal and patrilineal pattern often developed, with the male head of the household exchanging animals for wives and considering them—and the animals—part of the patriarchal possessions. Thus, a new social order was created based on these patriarchal values of obedience—of wife to husband, son to father, slave to master [5]—which also included the relationship with livestock.

In the Mediterranean region of the Fertile Crescent, where the domestication of animals and plants took on special importance [3], between 8000 and 3000 BC, male priestly and warrior elites developed, subjugating the populations of the surrounding villages. Tributary relations (the obligation to pay tribute to conquerors) and slavery developed over the conquered societies [8]. This period followed the time of the Hebrews. The Hebrews were a patriarchal society of shepherds who settled in Palestine in the final centuries of the second millennium BC. The God of the Hebrews was the creator of the earth and ruled over it, while humans were seen as separate from the rest of the animals, as they were made in God’s image. This meant that household heads shared God’s dominion over the animal and plant world by being the caretakers of the animals and plants of the earth.

Some time later, the Greeks, then a dominant society in this part of the world, proposed the superiority of human beings over animals, based on the existence of a “rational” soul present in humans and absent in animals. In fact, Greek culture was characterized by this search for the origin and destiny of the soul above the corporeal. In Aristotle’s writings on politics and the generation of animals [9], it is clear how the hierarchy of Greek male elites over slaves, women, and animals is based on a relationship of superiority of mind over body. Animals, like other relationships with people and objects, were viewed by these elites as a means of labor under their control and for their benefit. When Christianity arrived, it adopted both the Hebrew vision of human dominion over animals (exercised by the head of the household) and the Greek vision of the transcendence of the patriarch’s rational mind or soul over the body, women, and animals. This view persisted throughout the Middle Ages. For instance, Thomas Aquinas maintained the Aristotelian tradition by declaring that animals lack moral value because they lack rational souls. Consequently, humans could not only use animals for food and labor, but also recognized that the only reason not to mistreat them was that humans might become corrupt and learn to be cruel to each other by being cruel to animals [5]. In fact, in Western Europe during the Renaissance, animals were still considered to be immoral beings due to their lack of rational souls. A good example of this is Descartes’s postulates, according to which animals were mere automatons that felt no pain and were therefore completely exploitable, not only for food and labor, but also as subjects of scientific experiments [10]. During the 18th and 19th centuries in England, anti-animal cruelty movements emerged from a new urban-born middle class that did not connect with the rural peasants’ or the aristocracy’s (hunters’) view of animals. This was when the first organizations against animal cruelty emerged, such as the Society for the Prevention of Cruelty to Animals, which at the time was heavily focused on public spectacles, such as cockfighting and bullbaiting, which produced animal suffering [11]. These campaigns against animal cruelty were often closely associated with parallel campaigns against cruelty to prisoners and in favor of the abolition of slavery [5]. In Great Britain, laws were created to regulate potentially cruel practices toward animals as early as 1822 [11], although the most important of these laws was the Animal Protection Act of 1911. This act defined cruelty as both active and passive, that is, by omission and commission. It made it a crime to “hit, kick, maltreat, knock down, force, torture, enrage, or terrorize an animal” and to cause unnecessary suffering through any act [12]. However, the law applied only to public spaces, and therefore, no livestock owner, regardless of what they did to their animals, had ever been prosecuted under it [13].

## 3. Animal Welfare and the Beginning of Emancipated Values

In 1964, Ruth Harrison, a British author, animal welfare campaigner, and housewife who had received a campaign leaflet denouncing animal abuse on farms, published *Animal Machines* [13]. With exhaustive research based on consultations with scientific and professional experts, such as veterinarians, and using rigorous argumentation and a dispassionate tone, she used the book to denounce the detrimental effects of intensive farming on animals. She argued that livestock suffered various forms of cruelty that the 1911 Act neither prevented nor controlled. While they were able to continue growing and reproducing, they experienced “discomfort, boredom, and a veritable denial of health... the animal is not allowed to live before it dies” [13]. *Animal Machines* received enormous publicity. It was serialized in the *Observer* newspaper and widely reported on elsewhere [12]. Following pressure from the RSPCA, the government decided to set up an independent commission to address the question. To limit the possibility of this commission issuing uncomfortable recommendations, it was given a narrow, technical mandate: “to examine the conditions in which livestock are kept under systems of intensive husbandry and to advise whether standards ought to be set in the interests of their welfare, and if so, what they should be” [12]. A panel of independent scientific experts, chaired by zoology professor F.W. Rogers Brambell, was appointed to investigate this problem. Its official name was the “Technical committee to enquire into the welfare of animals kept under intensive livestock husbandry systems” [14]. Although there was no official justification or presentation of the term as such, the use of the term “welfare” in the Brambell Committee’s title and terms of reference was vitally important for its adoption. It should be noted that Ruth Harrison did not use the term “welfare” even once in her book, but rather used terms like suffering or cruelty, more in tune, in fact, with the tools available to society at the time, based on the aforementioned 1911 Anti-Cruelty Act. However, referring to the concepts of “cruelty” or “suffering” in the Brambell Committee’s title would have implied recognition that these problems could exist in intensive livestock farming promoted by the same governments in previous periods. By contrast, welfare—derived from an Old English term meaning to ‘fare well’—had more positive connotations relating to the capacity to grow and produce [12]. The association of the term “welfare” with the work of the Brambell Committee precipitated its incorporation into the general vocabulary. In fact, the report published in 1965 provides one of the first definitions of welfare: “a broad term encompassing both the physical and mental well-being of the animal” [14]. Therefore, while the adoption of the term welfare instead of cruelty may be partly a linguistic issue, it may also have had a strategic purpose [12]. After some years, in many areas where it was usual to talk about preventing cruelty to animals, the term was changed to animal welfare, becoming, with time, a major focus of political, ethical, and scientific attention. In fact, in the 1960s, the emphasis of discussions was on what people should do, i.e., on animal protection rather than on animal welfare. In the 1970s and early 1980s, the term “animal welfare” was used, but as Donald Broom recognized, it was not defined and not considered scientific by most scientists [15]. The work of various ethologists and psychologists of that time on animal motivation was fundamental to giving substance and meaning to the young concept of animal welfare. A review by Broom (1981) of a book entitled *Behavioral Biology* noted that the animals described therein were presented as sophisticated decision-makers in almost all aspects of their activity [16]. This perspective contrasts with the idea of animals as automatons driven by “instinct.” The research of authors such as Ian Duncan, Barry Hughes, and David Wood-Gush, which explained the biological bases of animals’ needs and the frustration that arises when these are not met, is just one example [17,18,19].

On the other hand, it is also true that Ruth Harrison was not the first person to denounce the practices carried out on intensive farms, nor did these practices occur only in the 1960s. But the book, apart from being well written, found a favorable environment for its promotion. In the 1960s, the United Kingdom experienced a cultural revolution that brought about new values described as emancipatory. The 1960s were years marked by protest, idealism, and rebellion, with a youth culture that set trends, changes in personal relationships and sexual behavior, underground and counterculture movements, the struggle for Black civil rights, a new feminism, gay liberation, and a new kind of popular music [20]. In other words, the beginning of a transition from patriarchal values (those that emerged with the rise of agricultural and livestock production) to emancipatory values, focused on a central concept—freedom. This contrasted with 1950s society, characterized by a very rigid social hierarchy; the subordination of women to men and of children to parents; repressed attitudes toward sex; racism; unquestioning respect for authority in the family, education, government, law, and religion; and strict formalism in language, etiquette, and dress codes [20]. Logically, for a conservative of those years, these were times of morality, patriotism, law and order, and respect for the family. It must be understood that this generation had lived through two world wars and the economic depression and poverty that followed. But all of this, thanks to the good work conducted by this previous generation, seemed very distant and irrelevant to the twenty-somethings of the 1960s, who questioned the need for those social and moral norms in their time, with a growing interest in individual liberties and personal expression. According to political scientists Ronald Inglehart and Christian Welzel, it is not the rationalization of authority but the emancipation of authority that becomes the dominant trend of modernization, transforming modernization into a process of human development that promotes human emancipation on all fronts [21]. The truth is that if there is a universal human aspiration, it is the right to free choice and autonomy. But when one’s own survival is at risk, these desires become a secondary priority. That is, in contexts where physical survival is not assured, the desire for physical and economic security tends to take priority over other values. It is when these basic needs (physiological and security) are met that other values, such as self-expression, are prioritized [21]. Emancipative values constitute the key cultural component of a broader process of human empowerment, changing people’s life strategies from an emphasis on securing a decent subsistence level to enhancing human agency, prioritizing lifestyle liberty, gender equality, personal autonomy, and the voice of the people [22]. These values, which advocate for the expansion of personal freedoms, self-expression, equality, and autonomy, have been theorized to enhance life satisfaction, primarily through fostering a growing sense of empowerment and creating a wide array of opportunities for individuals [23]. This empowerment manifests itself in various aspects of life, including the exercise of property rights, access to education, freedom of expression, greater interpersonal tolerance and trust, and active civic participation, all of which contribute to personal and social development [24]. All of this, including the desire for freedom, influences the way animals are viewed and treated, so it is no surprise that the Brambell Committee in the 1960s in the UK introduced the concept of the Five Freedoms when attempting to define the new concept of animal welfare.

## 4. The Five Freedoms

The Brambell Report articulated for the first time the concept of the Five Freedoms, consisting of the freedom to turn around, stand up, lie down, scratch, and stretch their limbs (referring to animals). At the same time, the report asked for the creation of a Farm Animal Welfare Advisory Committee, which is now known as the Animal Welfare Council (AWC). Considering the Five Freedoms as a foundational concept in animal welfare, this advisory committee refined them into something closer to the current concept, and defined them as freedom from hunger, thirst, or malnutrition; appropriate comfort and shelter; prevention or rapid diagnosis and treatment of injury and disease; freedom to display most normal patterns of behavior; and freedom from fear [25]. Given the 1979 definition, the first to detail the broader dimensions of animal welfare by incorporating subjective experiences, health status, and behavior [26], it was in the 1990s when the definitive Five Freedoms, which have remained virtually unaltered since then, were created [27]. These freedoms, which represent ideal states rather than actual standards for animal welfare, include freedom from hunger and thirst, freedom from discomfort, freedom from pain, injury, and disease, freedom to express normal behavior, and freedom from fear and distress (Table 1). They combine elements from three different approaches to welfare. This is the emotional state of the animal (distress, fear, and pain), the ability to show normal patterns of behavior (freedom to express normal behavior), and biological functioning (the rest). For years, they have been a very useful set of principles for identifying the main welfare problems as well as a starting point for identifying the main welfare components. However, in recent years, they have been complemented with other concepts, such as the Five Domains.

In 2004, the European Commission funded the largest scientific project on animal welfare ever funded. With a total budget of EUR 17 million, involving 17 countries and 40 research institutions, under the acronym Welfare Quality (WQ), one of the project’s objectives was to create animal-based assessment protocols for three species—cows, pigs, and chickens/laying hens. One of the first actions of this project was to define the concept of animal welfare. As mentioned, the Five Freedoms were extremely popular at the time in both scientific and non-scientific settings. However, these freedoms were deemed not precise enough to be used as the basis for welfare assessment. Then, within the Welfare Quality Project, the Five Freedoms were redefined in terms of four principles—good feeding, good housing, good health, and appropriate behavior. At the same time, these principles were developed under 12 criteria (Table 1).

For instance, in good feeding, we could find something like the first freedom, as it was defined as the absence of prolonged hunger and thirst. The second principle was also similar to the second freedom, but easier to implement; in this case, it is defined as comfort during resting, thermal comfort, and ease of movement. The third principle was directly extracted from the Five Freedoms, as it contained the absence of disease, pain, and injuries. Finally, the fourth principle contained two main terms from the Five Freedoms, namely, fear and the capacity to show normal behavior, with the latter translated as the expression of other behavior and social behavior, and included one very important concept not considered in the Five Freedoms, namely, the human–animal relationship (Table 1). The most interesting aspect is what happened from the beginning to the end of the Welfare Quality project, as the 12 criteria from 2005–2007 differed from those from 2008–2009. This was because society and its values were also changing during this period, and the protocols only addressed negative aspects to define the welfare of animals. As a consequence, the twelfth criterion of Welfare Quality, which in the first part of the project was called ‘absence of fear’, was changed in the last years of the project to ‘positive emotional state’. This affected not only the protocols developed within the framework of this project, but also all protocols developed under the Welfare Quality philosophy after its completion. A direct consequence of this change was the inclusion of the Qualitative Behavioral Assessment (QBA) parameter in all farm welfare protocols used in Welfare Quality. This parameter considers positive and negative emotions in animals [28] and is present in all three species [29,30,31].

## 5. From the Five Freedoms to the Five Domains and the Transition to the Knowledge Society

**Figure 1:**
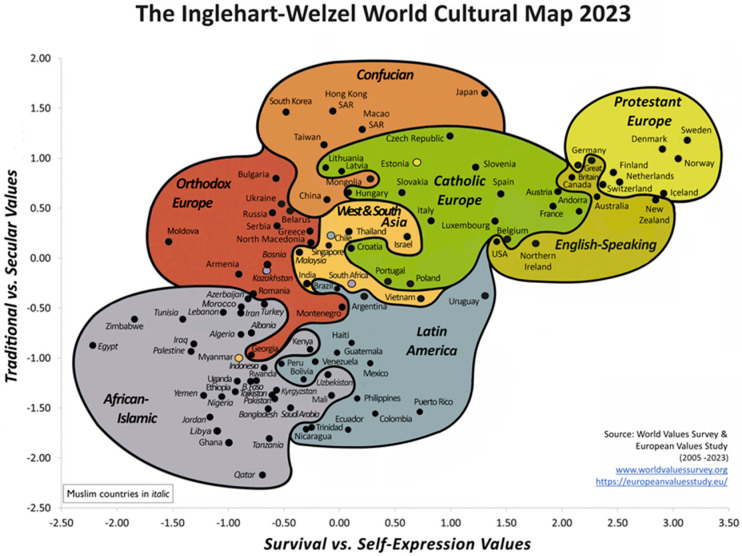
The Inglehart–Welzel World Cultural Map—World Values Survey 7 (2023). Source: http://www.worldvaluessurvey.org/ (accessed on 1 September 2025) [32].

Analysis of the World Values Survey (WVS) data made by political scientists Ronald Inglehart and Christian Welzel asserts that there are two major dimensions of cross-cultural variation in the world, as follows: (1) Traditional values versus secular-rational values and (2) survival values versus values of self-expression. This can be shown in a map that presents empirical evidence of massive cultural change and the persistence of distinctive cultural traditions (Figure 1). Moving upward on this map reflects the shift from traditional values to secular–rational, and moving rightward reflects the shift from survival values to values of self-expression. Traditional values emphasize the importance of religion, parent–child ties, deference to authority, and traditional family values. Secular–rational values societies place less emphasis on religion, traditional family values, and authority. Survival values place emphasis on economic and physical security. They are linked to a relatively ethnocentric outlook and low levels of trust and tolerance. Values of self-expression give high priority to environmental protection, growing tolerance of foreigners, gender equality, and rising demands for participation in decision-making in both economic and political life [21]. The World Values Survey (WVS) is an international research program devoted to the scientific and academic study of social, political, economic, religious, and cultural values of people throughout the world. The main research instrument of the project is a representative comparative social survey, which is conducted globally every 5 years. At the moment, WVS is the largest non-commercial cross-national empirical time-series investigation of human beliefs and values ever conducted.

According to the authors, cultural modernization could be based on a two-stage process. In the first phase, the industrial sector grows at the expense of the agricultural sector. This is linked with the rationalization of authority, reflected in rising secular–rational values. In the second phase, the service sector grows at the expense of the industrial sector. This transition from industrial to knowledge society produces the largest increase in individual agency [21]. In other words, as the percentage of the workforce in the service sector grows and the size of the industrial sector shrinks, a society’s belief system tends to shift from survival to values of self-expression [21].

Equality among human beings, a key element of human rights, has traditionally been approached from a purely rational perspective. However, its foundations are much more emotional than rational, and empathy plays a fundamental role in this. It is through empathy rather than pure reason that another person is perceived as equal (equal in the sense of being an equally sensitive being). The values of a knowledge society promote humans who are more emotionally connected by creating spaces where people feel free to express and experience their full range of feelings [21]. By promoting autonomy, people are empowered to make decisions that align with their true emotions rather than following rigid social norms that may suppress their emotional expression. By freeing themselves from social constraints, people can better explore and understand their own feelings, which in turn improves their ability to connect with the feelings of others. Furthermore, the different emotions are valued and respected, fostering empathy and compassion among people, making society more egalitarian and fairer. A more emotional society can have better treatment of animals due to the greater capacity for empathy and emotional connection that develops among its members. This empathy facilitates understanding of animals’ needs and emotions, leading to greater respect and care.

In 2007, the European Union officially considered animals as sentient beings, which means capable of feeling emotions. In fact, this notion was already included in the European Union Treaty of Amsterdam (European Union, 1997, p. 110), but it was not until the Lisbon Treaty that this became an official consideration in the EU [33]. This means that animals can no longer be considered machines that can be manipulated for human purposes. Because animals are sentient, their welfare matters [34], and scientists have argued that since emotional responses of some animals are influenced by cognitive processes, these animals not only experience emotional responses, but really do feel emotions [35]. Researchers further considered that if animals can use checks similar to humans, there was no reason to reject the idea that these animals could feel the emotions felt by humans because of the combined outcomes of these checks. As an example, the study by Veissier et al. (2009) on sheep concludes that sheep can experience a wide range of emotions, as follows: i) fear and anger, as they are sensitive to suddenness, unpredictability, controllability, and social norms; ii) rage, as they respond to suddenness, unfamiliarity, unpredictability, discrepancy from expectations, controllability, and social norms; iii) despair, as they react to suddenness, unfamiliarity, unpredictability, discrepancy from expectations, and controllability; and iv) boredom, as they are sensitive to suddenness, unfamiliarity, unpredictability, discrepancy from expectations, and controllability [36]. Furthermore, in the same year (2009) that Welfare Quality released its final outputs [29,30,31], the UK FAWC wrote an influential report on the future of farm animal welfare [25] in which the Five Freedoms were complemented with the concept of a Life Worth Living (LWL). The idea of a Life Worth Living is valuable for the animal, as it focuses on the animal’s feelings [37] and quality of life [25,38,39], which can be considered the result of its affective state over a period [40]. FAWC introduced the concept of a Good Life as well as the highest category, “over and beyond” that of an LWL. FAWC defined this concept as involving an especially high affective ratio of positive to negative experiences [25]. Therefore, in a short period of time, the EU officially recognized animals as sentient beings, Welfare Quality evolved its structure to define animal welfare, changing ‘absence of fear’ to ‘positive emotional state’ to include positive emotions, and the owner of the Five Freedoms (where mainly negative emotions, such as pain, fear and distress, are considered) proposed complementing the approach with the concepts of a Life Worth Living and Good Life. This is clearly not just a movement within science, but a movement of a science connected to the values of a society. In fact, scientists and society in the 1980s considered the Five Freedoms an excellent approach to animal welfare, and since then, they have been essential in the advancements made in this field. However, for a citizen of 2025, born into a highly emotional knowledge society, claiming that an animal is fine because it is not suffering distress, pain, or fear (the emotional states considered in the Five Freedoms) could be closer to the definition of an abuser that does not consider the valence of other individuals than someone truly concerned about animal welfare. In other words, for an emotional society, the statement: “if I don’t hit you, punish you, or terrorize you, your well-being is guaranteed” falls short. Therefore, the Five Freedoms alone, for this citizen, would not be enough nowadays, and need some kind of complementary approach, which is what the Five Domains provide. In fact, this view of animal welfare and this view of human rights, where the presence of emotions is essential, can explain why the concept of the Five Domains, published for farm animals for the first time in 2016 [27,41], was so successful in the scientific community. Although the concept could be argued to be a logical evolution of the 4 principles and 12 criteria of the Welfare Quality, the truth is that the same authors already suggested this concept in the 1990s for laboratory animals [42], so they provided the Five Domains before the creation of the Welfare Quality. However, society in the 1990s was probably not ready for this concept, while society in the 2020s is. The Five Domains establish that animal welfare is based on good nutrition (feeding in WQ), a good environment (housing in WQ), good health, and good behavior (appropriate behavior in WQ). Extracting the twelfth criterion from WQ as a separate domain, the fifth is mental state (positive emotional state in WQ). Furthermore, it explains that the first four domains culminate in a definitive fifth, mental state, which is what defines an animal’s welfare. This view, when sentience is acknowledged and mental state is placed at the center of the definition, means several things, one of the most important being that to study mental state, we need to study animals’ emotions; these include both positive and negative states. Therefore, a new concept emerges in which the absence of negative consequences for the animal can prevent poor welfare, but good or better welfare, also known as positive animal welfare, requires the study of positive indicators of animal welfare. This is, of course, in line with the concepts of an LWL and a Good Life of the AWC. In fact, although the focus has mainly been on negative aspects of welfare since the start of the modern animal welfare debate (post-1960s), there is increasing scientific acceptance of animals having the capacity to experience positive emotions [43]. Recently (2025), a scientific consensual definition for “positive welfare” has been provided [44]. Since Mellor (2016) defined the Five Domains as a refinement of the Five Freedoms [27], Webster (2016) defined them as complementary [45]. According to Webster (2016), while Five Domains seeks to assess the impact of the physical and social environment on the mental state of a sentient animal, Five Freedoms is an outcome-based approach used to identify and evaluate the efficacy of specific actions necessary to promote well-being [45]. Therefore, for a citizen of 2025, born into a highly emotional society as described above, both complementary approaches could be used for different objectives [45].

While this was happening in how animal welfare was defined and seen, more researchers were using the Welfare Quality approach to assess animal welfare [46]. This approach involved not only the use of the 4 principles or 12 criteria, but the use of animal-based indicators. This is based on the fact that, since welfare is a condition of the individual animal, wherever possible, it emphasizes animal-based measures (also called ‘outcome’ or ‘performance’ measures) rather than resource and management inputs, in an attempt to estimate the actual welfare state of the animals. Such physiological, health, and behavioral measures have inherent advantages over input “resource” measures. The first advantage is clearly that, since welfare is a condition of the animal, outcome measures are likely to be the most direct reflection of its actual welfare state. It permits evaluating the welfare by directly observing the animal, regardless of how and where it is living. Secondly, it is applicable to any production system, as, for instance, an animal with lameness or one that exhibits positive social behavior will show the same signs in one country or production system as in another, and will remain more transparent to stakeholders worldwide. Animal-based measures are evaluative, obtained in a precise way, and usually quantitative. They can be collected on-farm either by observation or inspection of the animal or by assessing the effects of a response on the environment. For example, loose feces on the floor are evidence of diarrhea in a group. In relation to that, it is important to consider three aspects—validity, repeatability, and feasibility. Validity is defined as the extent to which we can actually measure what we are supposed to, or, in other words, the extent to which a measure is meaningful in terms of providing information on the welfare of an animal or a group of animals. Repeatability is defined as the similarity of repeated measurements on the same item (e.g., farm or group of animals). Concerning on-farm welfare assessment, repeatability generally has two components—inter-observer and test–retest repeatability [47]. High inter-observer repeatability is achieved when two (or more) people carrying out an observation of the same animals report similar scores. Test–retest repeatability refers to the chance that the same results will be obtained if the test is repeated. In the special case of welfare assessment, the intra-observer repeatability—which describes the extent to which a single assessor obtains consistent results when evaluating the same animals or farms on different occasions—is part of test–retest repeatability. Test–retest or intra-observer repeatability of measures is usually recorded over a short lapse of time between observations, or the same situation is assessed repeatedly from video clips [48]. A variety of methodologies have been used to determine repeatability. In animal welfare science, repeatability of continuous variables is often expressed as a Spearman or Pearson correlation coefficient [49,50]. In studies where three or more observers measure the same parameter [51] or where consistency over time is assessed more than twice [52], Kendall’s coefficient of concordance is calculated to quantify the overall reliability. The consistency of a test measurement can also be quantified by means of intra-class correlation coefficients (ICCs). In some cases, a measure is so difficult to harmonize or to train that it is not possible to obtain good repeatability between observers. In fact, harmonized definitions of ABMs are key factors to secure inter-observer repeatability. When this is not possible, although the parameter could be considered valid, it is not acceptable for benchmarking. The third point to take into account is feasibility. In this case, it is important to take into account the scenario in which the assessment will be performed. In any case, a distinction between animal protection (what people are allowed to do to animals) and animal welfare (probing the animal’s own experience of its situation) with validated measures has grown, and it is now accepted that animal welfare science is largely about the assessment of the animal’s own experience [53].

## 6. Discussion

The Welfare Quality philosophy, its four principles and twelve criteria, and the fact that animal welfare must be assessed using animal-based measures, have been incorporated into the general animal welfare frameworks of several schemes, including European institutions such as the European Commission and EFSA [54]. Furthermore, the success of this approach in the scientific world is demonstrated by the large number of animal welfare assessment protocols developed in recent years for species other than those covered in the original project, including small ruminants, horses, turkeys, ducks, dolphins, zoo animals, rabbits, dogs, quails, sea bream, sea bass, minks, foxes, and Finnish raccoons [55,56,57,58,59,60,61,62,63]. However, the transfer of all this knowledge, and even the basis of this knowledge, failed, in most cases, to reach the market. According to the 2022 EU study on animal welfare labeling, which compared 28 animal welfare certification schemes, one used only animal-based measures, while in the rest, most indicators were resource-based measures [64]. In the same study, the presence of mutilations was mentioned as an example of an animal-based measure included in most of these schemes, which is a management-based measure that can be taken simply by interviewing the farmer, without monitoring the animals. Monitoring systems and certification schemes rely heavily on examining inputs, i.e., “what” or “how much” different resources are provided to animals (e.g., pen design, outdoor access, enrichment material, space requirements, etc.). These measures have often been criticized for their low potential validity due to their indirect nature and complex interactions with other resources and management conditions [65]. In fact, input measures are an insufficient guarantee of animal welfare, as animals may experience the same situation or management procedure differently depending on their genetic origin, temperament, or previous experiences. For example, the term “free-range” could be defined solely in terms of grazing space or as a marketing “label” that attracts consumers to a program that, in reality, encompasses a more holistic approach. Furthermore, different consumers may have different opinions about what free-range means [36], and of course, two different farms offering the same type of free-range production may obtain very different results when the animals are assessed using a set of animal-based measures. It is true that there are many inputs (risk factors) that have the potential for improving animal welfare, but a certification system based on these risk factors is overlooking the most important factor of all: the animal. In this case, what is being assessed is human decisions, not the condition of the animals. When the answer to the question of what animal welfare is becomes caring for animals, the focus of the question, which was the animal, shifts to the human in the answer, “what we, as humans, are doing with animals”. This reflects an anthropocentric view typical of a patriarchal society, in which the needs of children, wives, and animals are met solely by the actions of the patriarch (e.g., now, you are fine, because I have given you this). Human decisions are undoubtedly easier to evaluate and communicate to society. For example: “Our system does not allow animals to be tied up, it is prohibited to dehorn cows, and we require 365 days a year with a minimum of 18 hours a day of access to pasture.” Of course, there is potential here to improve welfare, but the actual condition of the animals is not assessed, so the impact of these measures (or others) on the actual population is based solely on research conducted on another animal population and, in the worst case, even on mere speculation. For this reason, these human decisions should be validated by animal-based measures. It is true that many consumers seem to evaluate welfare based on the production system [66], in particular, on freedom [67]; naturalness [66]; the avoidance of certain mutilations [25]; and humane killing [37]. However, scientists highlight that greater freedom or naturalness could lead to competition, starvation, and predation [68,69], and that inter-individual differences may cause some animals to find a particular stimulus aversive rather than rewarding, influenced by their previous historical experiences and subsequent learning [37]. Consumers often confuse the concepts of freedom and naturalness, but for an animal living in a natural environment without access to sufficient food or water, there is no real freedom of choice. Consumer education and public education are key factors to consider. One of the problems lies in the fact that between animal welfare science and the consumers themselves, there are several intermediaries, starting with producers, industry, and retailers. All of these stakeholders have their marketing departments interested in conveying a specific message based on their own interests, and at the end of the chain, there is a consumer who typically spends only a few seconds making their purchasing decision. Alongside these actors are other stakeholders, such as NGOs, also with their own message, or the authorities, who also have their own communications departments and interests. In this context, it is essential to find ways to reach these citizens and explain to them that there is a science of animal welfare, and show them how it can be applied, for example, in animal welfare certification schemes. This is a task that animal welfare scientists should do as an essential part of their work and not delegate to other actors. A society educated in science will always be better equipped to find ways to overcome the aforementioned barriers and access the science that should lie behind every recommendation or label. The mere existence of such consumers will also encourage other stakeholders to seek messages that are more grounded in science. Ultimately, not only will the system be more robust and less prone to greenwashing, but consumers themselves will be better able to detect it, and putting the animal in focus with animal-based measures is a first desirable step.

In any case, in recent years, there has been a common strategy on the part of retailers, producers, and NGOs to bring animal welfare to the market, not to check the animals, but to check the facilities and management. For retailers, this is something easy to communicate to consumers, sometimes with a rating system based on stars, letters, or numbers. For producers, it is also a simpler system, as a quick checklist allows them to see where they stand and even what expenses they must incur to move from one level to another. In fact, according to the EU study on AW labeling, the fact that most schemes use basically resource-based measures is because they are easier to check and communicate [64]. For NGOs, in the words of the Eurogroup for Animals platform, which brings together Europe’s leading animal welfare NGOs, “Incorporating information about the way in which animals are reared (method of production) on the labels of animal-sourced products has the potential to transform consumption patterns, which can contribute to improved levels of animal welfare on farms. Method-of-production labeling on animal-sourced products helps consumers support production processes that are more respectful of animal welfare. In turn, this can accelerate a transition to a more humane animal agriculture industry.” In fact, it is not a bad strategy, as demonstrated by the case of egg labeling for laying hens in Europe in 2008. Since then, the number of laying hens raised in alternative, cage-free systems has increased dramatically in the EU, in most cases because retailers stopped selling eggs from caged hens.

Of course, although retailers, producers, and NGOs have different objectives when using inputs, a production-system classification, or simply good practice guidelines to certify animal welfare, it could be argued that they are only trying to meet public expectations, which must be taken into account because animal welfare is a social concept [70]. In fact, we have seen how the definition of animal welfare has evolved with the evolution of society and its values. We can also argue that animal welfare labels should be relevant to consumer concerns, as it is consumers who must make purchasing decisions and pay for improvements in welfare [37]. However, in a society where values such as equality are linked to personal freedoms, self-expression, autonomy, and empathy, it is only a matter of time before animal welfare certification labels that only take into account inputs—that is, what humans provide to animals—but not the actual life experiences of animals in those environments—are considered insufficient. According to Vanhonackewr et al. [71], schemes should translate scientific definitions of animal welfare into a “popular” concept, and this is an effort that must be undertaken by scientists. The concept of the Five Domains or LWL and the Good Life or Positive Welfare, or even the Welfare Quality approach, which includes positive emotional states in its twelve criteria, all point in the same direction. According to Yeates [37], an animal welfare certification program should consider the concepts of LWL and Good Life, combining an outcome-assured and production-based approach that combines consumers’ opinions and outcome-based animal welfare measures. This could be a good solution; as mentioned above, consumer perception is important and must be taken into account, but it should not be sufficient to obtain an animal welfare certificate. A document certifying animal welfare should consider the animals and their life experience on each farm, without assuming that all farms classified in a specific category based on consumer concerns (or those of NGOs or producers) have exactly the same outcome in terms of the quality of life of the animals living on them. It is unfair to the animals not to take them into account in the equation. It could be argued that one possible solution is to include some animal-based measures in a resource- and management-based (input) checklist. This is still unfair to animals because, as explained above, we already have science-based assessment protocols that provide a way to assess animal welfare from a multidimensional approach. Therefore, one day, the first step before creating a certification system will be to determine what the ultimate goal is. Is the objective to guarantee production methods (inputs) or to guarantee animals’ experienced quality of life (outcomes)? In the last case, the assessment focuses on animals, and in the first, it focuses on what affects animals: the environment and management. Probably the best approach is a combination of both. In fact, this is a model that already exists. When a person books a hotel in Barcelona, New York, or Paris, they obtain three types of information: the price, the star rating, and user reviews. Hotels earn star ratings from third-party organizations and tourism bodies that conduct inspections based on a predefined set of criteria, which generally include factors like room quality, staff professionalism, service efficiency, cleanliness, amenities, and security measures (most of them being inputs). The rating process involves submitting detailed documentation and undergoing on-site audits to verify that the hotel meets the standards for the desired star level, but real users are not asked to rate the hotel within the star system based on their own experiences. The inputs provided by the hotels of different categories (stars) will vary in different cities, as they depend on local agreements between stakeholders, as is the case with most local animal welfare certification systems currently in place in different countries [64]. They are based on inputs and are agreements between producers and administration, retailers and producers, NGOs and producers, etc. [64]. In contrast, the score given by people who have used the hotel (usually a score from 0 to 10 given on specific platforms) refers to their actual experience at these facilities, similar to how animal welfare assessment protocols developed and validated by science could be applied. The combination of these two approaches, a classification of the production system to connect with consumer expectations and the application of an animal-based protocol farm to farm to empower animals and take into account their life experience, will one day be considered the only fair approach. Just as the Five Freedoms, in a knowledge society in 2025, may be perceived as insufficient and need to be complemented with something like the Five Domains, one day, an animal welfare certification system that does not consider the actual life experience of animals through the use of protocols focused on animal-based measures will be considered unacceptable.

## 7. Conclusions

The market is dominated by animal welfare certification schemes that focus on inputs (what humans provide or the management practices they apply) rather than outcomes (animal-based indicators), from an anthropocentric perspective of animal welfare that does not consider the actual experience of animals from farm to farm. NGOs, producers, and retailers, based on different interests, agree to defend this approach, which is based more on a classification of the production model than on a real assessment of animal welfare, due to the ease of connecting the label with consumer expectations. However, in recent decades, scientists have developed several tools, such as animal-based assessment protocols, to consider the life experience of animals. In a knowledge society, it will soon be mandatory to empower animals by asking them how they are, rather than accepting that welfare is defined through input-based checklists. It is impossible to be fair to animals if they are not considered (using animal-based indicators) in an animal welfare assessment protocol. Therefore, it is expected that in the future, in these emotional knowledge societies, we will see schemes that combine a classification of the production system (inputs) and a real assessment of the quality of life of animals on each farm (outputs).

## Figures and Tables

**Table 1:** Relationship between the four principles and twelve criteria of the Welfare Quality project and the Five Freedoms of animal welfare (including the change of the 12th criterion after 2008).

Welfare Quality Principles	Welfare Quality Criteria	Five Freedoms
Good Feeding	Absence of prolonged hunger	Related to Freedom from hunger and thirst
	Absence of prolonged thirst	
Good Housing	Confort around resting	Related to Freedom from discomfort
	Thermal confort	
	Ease of movement	
Good Health	Absence of injuries	Related to Freedom from pain, injury or disease
	Absence of diseases	
	Absence of pain induced by management	
Appropriate Behaviour	Social Behavior	Related to Freedom to express normal behaviour
	Other behaviors	Related to Freedom to express normal behaviour
	Human animal relationship	Not directly related to any freedom
	Absence of fear (2004–2007)	Related to Freedom from fear and distress
	Positive emotional state (since 2008)	Not directly related to any freedom

## Data Availability

No new data were created.

## Abbreviations

The following abbreviations are used in this manuscript:

EU	European Union
FAWC	Farm Animal Welfare Council
ICC	Intra-class correlation coefficient
LWL	Life Worth Living
NGO	Non-governmental organization
QBA	Qualitative Behavioral Assessment
WQ	Welfare quality
WVS	World Values Survey
